# Supplementary figures and images for: Intracellular defensive symbiont is culturable and capable of transovarial, vertical transmission
Source: mBio. 2024 May 7;15(6):e03253-23. doi: 10.1128/mbio.03253-23 (PMC11237597; doi:10.1128/mbio.03253-23)

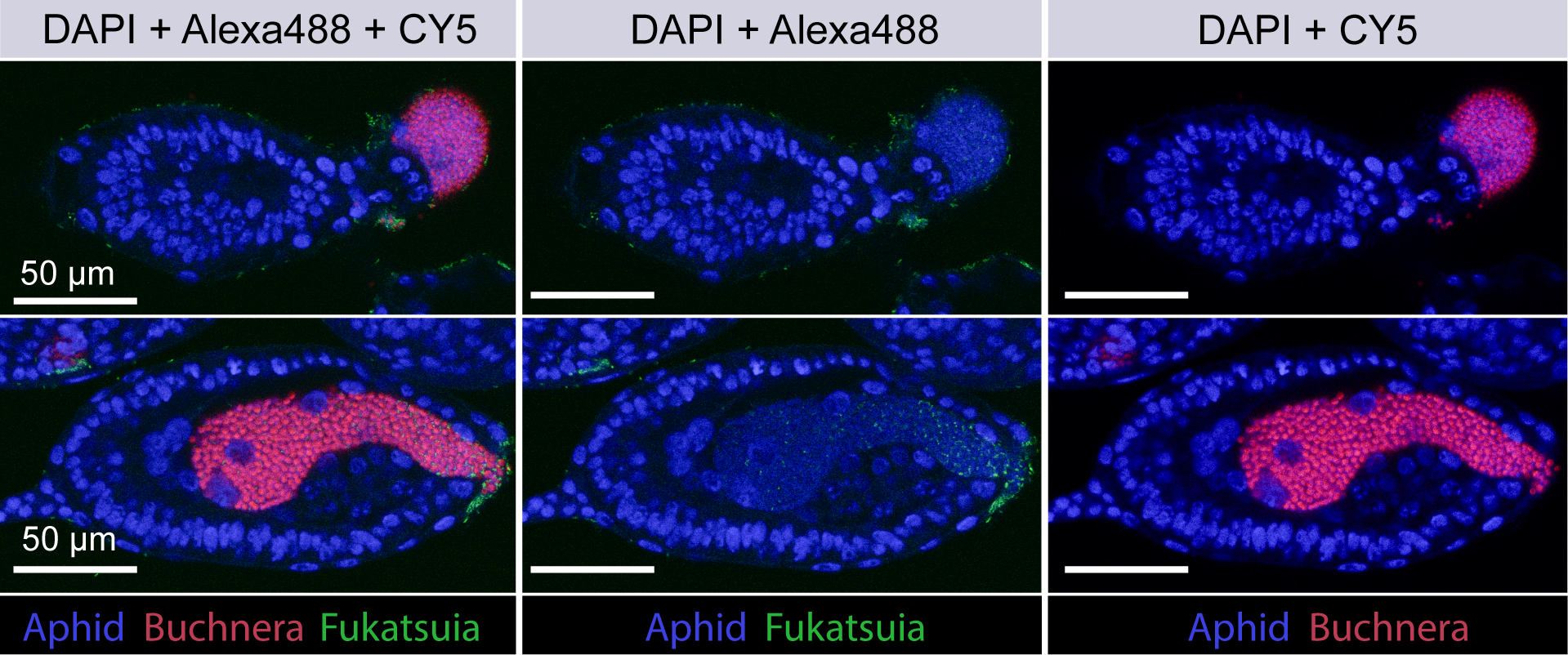

Supplement: Fig. S1 — Two-channel images of Fig. 4A and B. [file mbio.03253-23-s0001.tif]

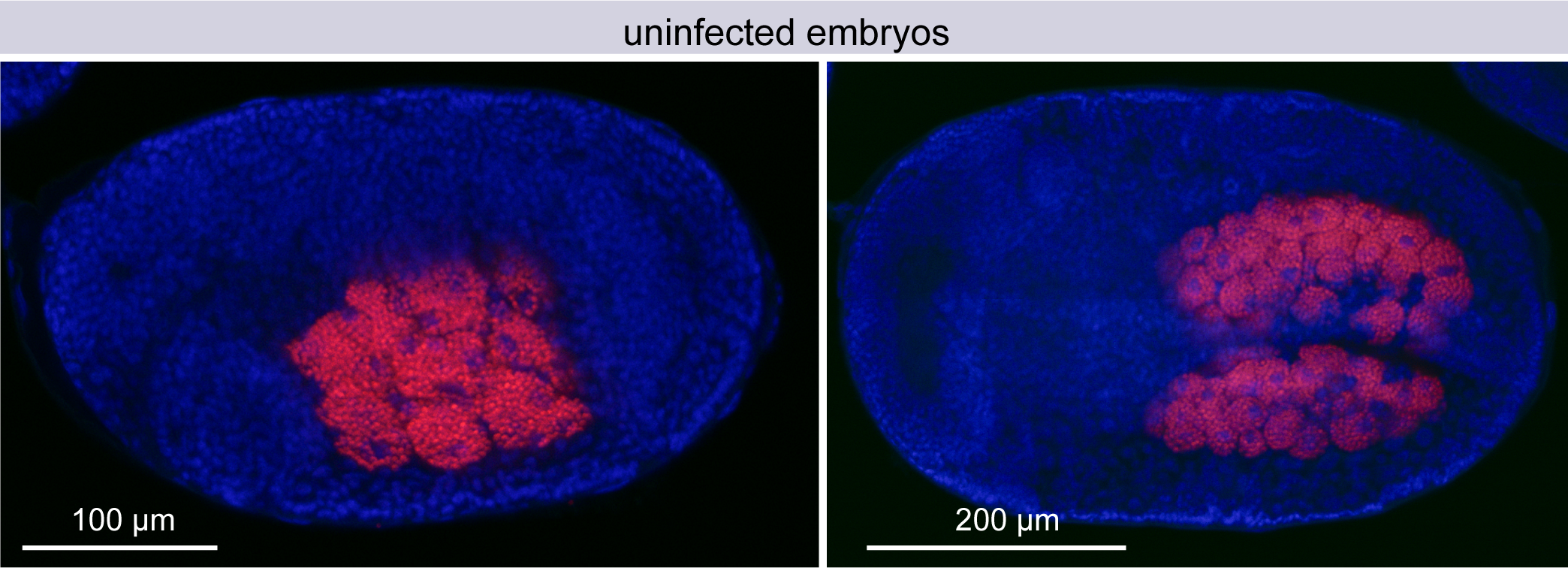

Supplement: Fig. S2 — Control images of aphid embryos not infected with Ca. F. symbiotica. [file mbio.03253-23-s0002.tif]

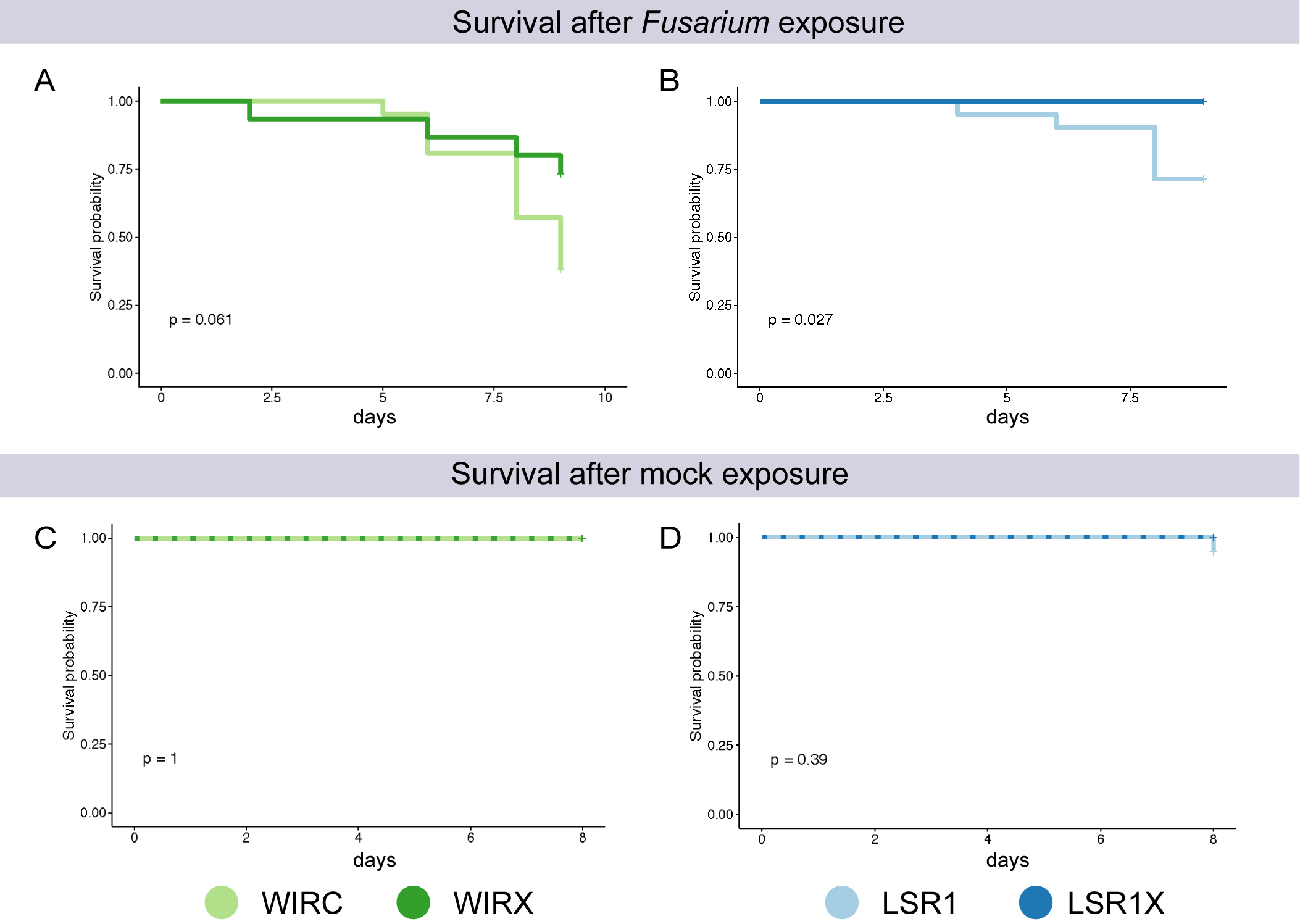

Supplement: Fig. S3 — Kaplan-Meier survival curves of aphids. [file mbio.03253-23-s0003.tif]
