# Supplementary material for: Intracellular defensive symbiont is culturable and capable of transovarial, vertical transmission
Source: mBio. 2024 May 7;15(6):e03253-23. doi: 10.1128/mbio.03253-23 (PMC11237597; doi:10.1128/mbio.03253-23)
Supplement: Legends — Supplemental legends. [file mbio.03253-23-s0004.docx]

**Supplemental Figures and Tables**

**FIG S1** Two-channel images of Fig. 4A and B.

**FIG S2** Control images of aphid embryos not infected with *Ca*. F. symbiotica. Embryos were treated with the same hybridization solution as in Fig. 4, containing probes targeting *Ca.* F. symbiotica, *Buchnera*, and DAPI. Control embryos were imaged with the same exposure settings as in Fig. 4.

**FIG S3** Kaplan-Meier survival curves of aphids after exposure to entomopathogenic *Fusarium* (A-B) and after mock treatment with 0.5% TWEEN80 (C-D).

**Table S1** Genome assembly statistics for *Ca*. F. symbiotica WIR.

**Table S2** Genome accessions for strains used in Fig. 2.
