# Supplementary material for: Intracellular defensive symbiont is culturable and capable of transovarial, vertical transmission
Source: mBio. 2024 May 7;15(6):e03253-23. doi: 10.1128/mbio.03253-23 (PMC11237597; doi:10.1128/mbio.03253-23)
Supplement: Supplemental tables — Tables S1 and S2 [file mbio.03253-23-s0005.docx]

| **Table S1. Genome assembly statistics for *Ca.* F. symbiotica WIR.** | | | | |
| --- | --- | --- | --- | --- |
| strain | WIR |  |  |  |
| total size | 3,065,463 |  |  |  |
| GC | 43.77% |  |  |  |
| Contigs | 51 |  |  |  |
| N50 | 1,916,902 |  |  |  |
| L50 | 1 |  |  |  |
| N75 | 258,911 |  |  |  |
| L75 | 3 |  |  |  |
| Completeness | 98.59% |  |  |  |
| Contamination | 0.19% |  |  |  |
| Illumina Read Pairs | 2,775,760 |  |  |  |
| Illumina Bases | 822,603,373 |  |  |  |
| Illumina Bases > Q30 | 780,675,249 |  |  |  |
| Nanopore Reads | 150,000 |  |  |  |
| Nanopore Bases | 639,543,275 |  |  |  |
| coverage depth | 463.30 |  |  |  |

| **Table S2. Genome accessions for strains used in Figure 2.** | | |
| --- | --- | --- |
| Organism | Strain | Assembly |
| *Ca*. Fukatsuia anoeciicola | Ancorni | <https://doi.org/10.5281/zenodo.7324976> |
| *Ca*. Fukatsuia anoeciicola | Anoenotherae | https://doi.org/10.5281/zenodo.7324976 |
| *Ca*. Fukatsuia symbiotica | Drplantanoidis | https://doi.org/10.5281/zenodo.7324976 |
| *Ca.* Fukatsuia symbiotica | WIR | this publication (JAXAWC000000000) |
| *Escherichia coli* | K12 | GCF_000005845.2 |
| *Ca*. Regiella insecticola | LSR1 | GCF_000143625.1 |
| *Serratia symbiotica* | Tucson | GCF_000186485.1 |
| *Yersinia pestis* | A1122 | GCF_000222975.1 |
| *Ca*. Hamiltonella defensa | MED | GCF_000258345.2 |
| *Ca*. Regiella insecticola | 5.15 | GCF_000284655.1 |
| *Ca.* Sodalis pierantonius | SOPE | GCF_000517405.1 |
| *Sodalis praecaptivus* | HS1 | GCF_000517425.1 |
| *Photorhabdus luminescens* | DSM 3368 | GCF_001083805.1 |
| *Ca*. Hamiltonella defensa | MEAM1 | GCF_002285855.1 |
| *Ca*. Hamiltonella defensa | A2C | GCF_002777195.1 |
| *Ca*. Hamiltonella defensa | ZA17 | GCF_002777235.1 |
| *Ca*. Fukatsuia symbiotica | 5D | GCF_003122425.1 |
| *Serratia marcescens* | KS10 | GCF_003516165.1 |
| *Arsenophonus nasoniae* | FIN | GCF_004768525.1 |
| *Ca*. Regiella insecticola | Tut | GCF_013373955.1 |
| *Xenorhabdus nematophila* | SII | GCF_014295015.1 |
| *Yersinia enterolitica* | NW56 | GCF_025758635.1 |
| *Ca*. Fukatsuia symbiotica | FuCiconfinis | GCF_900128755.2 |
| *Ca*. Hamiltonella defensa | HaCicuneomaculata-2628 | GCF_902859575.1 |
| *Ca*. Hamiltonella defensa | HaCiconfinis-2801 | GCF_902859675.1 |
